# Effect of Cu F Co-doping on the Properties of AgSnO_2_ Contact

**DOI:** 10.3390/ma12142315

**Published:** 2019-07-19

**Authors:** Jing-qin Wang, Zhou Liu, Ling Chen, Shuang-miao Yu, Yan-cai Zhu

**Affiliations:** 1 State Key Laboratory of Reliability and Intelligence of Electrical Equipment, Hebei University of Technology, Tianjin 300130, China; 2 Laboratory of Electromagnetic Field and Electrical Apparatus Reliability of Hebei Province, Hebei University of Technology, Tianjin 300130, China

**Keywords:** AgSnO_2_ contact, SnO_2_, Cu F co-doping, first-principles method, sol-gel method, wettability, electrical properties, mechanical properties

## Abstract

The crystal structures, mechanical properties, and electrical properties of Cu doped SnO_2_, F doped SnO_2_, and Cu F co-doped SnO_2_ were studied by using the first-principles method. Meanwhile, AgSnO_2_, AgSnO_2_-F, AgSnO_2_-Cu, and AgSnO_2_-Cu-F contacts were prepared by using the sol-gel method for a series of experiments to verify the theoretical analysis. According to the XRD patterns, the doping does not change the structure of SnO_2_, but increases its lattice constant and volume. Compared with the single-doped system, the doping formation energy of Cu F co-doped system is the smallest and the structure is more stable. Among the three groups of doping systems, the Cu F co-doped system has the highest shear modulus, Young’s modulus, hardness, and Debye temperature, and its mechanical properties and wear resistance are relatively best, and the melting point is also the highest. Cu F co-doping can further narrow the band gap of SnO_2_, reduce the electron effective mass and donor ionization energy, increase the electron mobility, and further enhance the conductivity of SnO_2_. The wetting angle of SnO_2_-Cu-F sample with Ag liquid is 1.15°, which indicates that Cu and F co-doping can significantly improve the wettability of SnO_2_ and Ag liquid. AgSnO_2_-Cu-F contact has a hardness of 82.03 HV, an electrical conductivity of 31.20 mS⋅m^−1^, and a contact resistance of 1.048 mΩ. Cu F co-doping can improve the shortcomings of AgSnO_2_ contact properties.

## 1. Introduction

In recent years, AgSnO_2_ contact material has attracted more and more attention due to its good welding resistance, arc ablation resistance, wear resistance, non-toxic and harmless properties, etc., and gradually replaced AgCdO contact material in low-voltage electrical appliances [1]. However, the contact resistance and temperature rise will increase during the operation of the AgSnO_2_ contact, which affects its electrical performance. Secondly, the high hardness and brittleness of the SnO_2_ makes it difficult to process the AgSnO_2_ contact material in the later stage. These problems greatly limit the application and development of the AgSnO_2_ contact material [2]. As a reinforcing phase, SnO_2_ directly affects the properties of AgSnO_2_ composites, so the properties of AgSnO_2_ composites can be improved by improving the properties of SnO_2_. In this study, the conductivity and toughness of SnO_2_ are improved by doping, and the wettability between SnO_2_ and Ag is also improved, which prevents SnO_2_ from precipitating out of the Ag liquid to form an enrichment zone to reduce the contact resistance of the AgSnO_2_ contact. Compared with the traditional powder metallurgy process, the SnO_2_ particles prepared by sol-gel method are small and can be uniformly distributed in the Ag matrix, which can improve the processing performance of the AgSnO_2_ contact material. Studies have shown that doping with Cu can effectively improve the wettability of SnO_2_ and Ag liquid, strengthen the bonding strength between Ag and SnO_2_ interface, and improve the ductility of AgSnO_2_ contact material [3]. Xu Jian studied the effect of F doping on the electronic structure of SnO_2_, indicating that doping F atoms can improve the electrical conductivity of SnO_2_ [4]. According to the co-doping theory proposed by Yamamoto and Katayama [5], the co-doping of two or more elements can further improve the properties of materials, therefore, it is necessary to study the properties of SnO_2_ and AgSnO_2_ contact after Cu and F co-doping. In this study, the first principles method was used to theoretically analyze the properties of different doping systems, meanwhile, the sol-gel method was used to prepare AgSnO_2_ contacts with different doping conditions, and their hardness, conductivity, and contact resistance were measured to verify the theoretical analysis.

## 2. Models, Calculation Method, and Experiment

### 2.1. Models and Calculation Method

**Figure 1:**
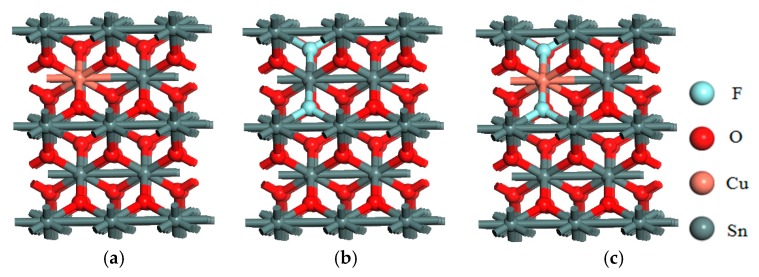
Doping models: (**a**) SnO_2_-Cu; (**b**) SnO_2_-F; (**c**) SnO_2_-Cu-F.

SnO_2_ is a tetragonal phase rutile structure, belongs to the body-centered tetragonal system, the space group is 136P4/MNM, the symmetry is D4H-14, and the lattice constants are a = b = 0.4737 nm, c = 0.3186 nm, α = β = γ = 90° [6]. The 1 × 2 × 2 supercell used in this paper contains 8 Sn atoms and 16 O atoms, which is obtained by expanding the unit of SnO_2_ in the y and z directions. The doping is realized by using the method of atomic substitution. The Sn atom located at the center is replaced by a Cu atom to realize Cu single doping, as shown in Figure 1a. Two F atoms are used to replace the O atoms closest to and next to Sn atoms for F single doping, as shown in Figure 1b. The Cu F co-doped model is shown in Figure 1c.

The Castep module (Cambridge Serial Total Energy Package) in the Materials Studio software based on Density Functional Theory was used for simulation calculation, the ultrasoft pseudopotential was used to describe the interaction between valence electrons and ionic solids, which can reduce the number of planar waves, using the PBE (Perdew-Burke-Ernzerhof) of GGA (Generalized Gradient Approximation theory) to deal with the exchange correlation functional, BFGS (Broyden Flecher Goldfarb Shanno) algorithm was used to optimize the models [7]. After the convergence test, the Ecutoff was set as 450 eV, and the k spatial grid point of brillouin region was set as 5 × 5 × 5. The convergence accuracy was 1.0 × 10^−6^ eV/atom, the stress deviation was not greater than 0.05 GPa, the interatomic force was less than 0.03 eV/Å, and the total energy convergence of each atom was set as 1.0 × 10^−5^ eV/nm in the calculation process. The valence electron configurations considered for each atom are: F: 2s^2^2p^5^, O: 2s^2^2p^4^, Cu: 3d^10^4s^1^, Sn: 5s^2^5p^2^.

### 2.2. Preparation of Samples

In this study, contacts were prepared by using sol-gel method and powder metallurgy method. The Cu F co-doped AgSnO_2_ contact is easy to be prepared, and the specific process is as follows: weigh a certain amount of SnCl_4_⋅5H_2_O and CuCl_2_·2H_2_O with a DT200A digital balance, and dissolve them in an ethanol distillation aqueous solution with a volume ratio of 1:1, and then add a certain amount of NH_4_F to the mixed solution. The solution was uniformly stirred by using the S21-2 type thermostatic magnetic stirrer, after 20 min, add ammonia water with a mass fraction of 10% in drops by drops. At the beginning, some white precipitate appeared, but the precipitate dissolved quickly. Continue to add a ammonia until the pH became 7, and the solution became all gelatinous. After standing and aging for 24 h, the colloidal solution was washed and aged to remove chloride ions by using an ethanol solution and a GL-20B centrifuge, the above process was repeated three times to obtain Cu F co-doped SnO_2_ gel. The Cu F co-doped SnO_2_ gel was dried in a WYK-11030 vacuum drying oven (120 °C, 1 h), and the dried powder was placed in a KSW-5-12A box type resistance furnace for sintering (500 °C, 1.5 h), and then grind and screen the powder through the WQS shake screen to get the Cu F co-doped SnO_2_ powder we need. The preparation process of Cu doped SnO_2_ powder and N doped SnO_2_ powder is similar to that of Cu F co-doped SnO_2_ powder. 

The doped SnO_2_ powder was mixed with Ag powder in a certain proportion, and the powder was fully mixed with QXQM-2 high-energy ball mill (the mixing time was 2 h, the forward rotation speed was 500 r/min, and the reverse rotation speed was 600 r/min). The mixed powder was subjected to initial pressure (25 T, 10 min), initial calcination (500 °C, 2 h), recompression (35 T, 10 min), re-firing (800 °C, 2 h), and finally polished with DMP-4A10 metallographic grinding and polishing machine to obtain Cu F co-doped AgSnO_2_ contact. The dimensions of all the samples prepared were the same, with a thickness of 3.5 mm and a diameter of 18 mm.

## 3. Simulation Analysis

### 3.1. Crystal Structure and Stability

The lattice constant, volume, and doping formation energy of SnO_2_ before and after doping are shown in Table 1. It can be seen from the table that the lattice constant and volume of SnO_2_ increase after doping. Firstly, after the doping atoms are incorporated into the SnO_2_ crystal, the differences in properties change the arrangement rule of the atoms, causing lattice distortion and expansion. Secondly, the introduction of F^−^ ions leads to an increase in the number of free electrons in the doping system and an increase in the repulsion, these two factors together lead to an increase in lattice constant and volume. At the same time, the increase of the crystal lattice will cause the position of the diffraction peak to move to a low angle, therefore, the angle corresponding to the four diffraction peaks of SnO_2_ after doping is slightly reduced, which is consistent with the X-ray diffraction experimental results.

The doping formation energy can characterize the difficulty of atom doping; the smaller the value, the easier the atom doping and the more stable the structure. The doping formation energy of the single doping systems are calculated as follows [8]:

(1) $$E_{f}\;(F)=E_{\mathrm{tot}}-E_{{\mathrm{SnO}}_{2}}+\mu_{O}-\mu_{F},\;E_{f}\;(\mathrm{Cu})=E_{\mathrm{tot}}-E_{{\mathrm{SnO}}_{2}}+\mu_{\mathrm{Sn}}-\mu_{\mathrm{Cu}}$$

The formula for calculating the doping formation energy of the co-doped system is:

(2) $$E_{f}\;(\mathrm{Cu},F)=E_{\mathrm{tot}}-E_{{\mathrm{SnO}}_{2}}+\mu_{O}+\mu_{\mathrm{Sn}}-\mu_{F}-\mu_{\mathrm{Cu}}$$

where, *E*_tot_ is the total energy of the doping system, *E*_SnO_2__ is the total energy of the SnO_2_ whose size is same with the doping system, $\mu_{F},\mu_{\mathrm{Sn}},\mu_{O},\mu_{\mathrm{Cu}}$ are the chemical potentials of the corresponding atoms. According to the Table 1, the doping formation energy of the Cu F co-doped system is smaller than that of the Cu single-doped system and the F single-doped system, indicating that Cu F co-doping is easier to achieve and the structure is more stable.

### 3.2. Electronic Structure

#### 3.2.1. Energy Band Structure

**Figure 2:**
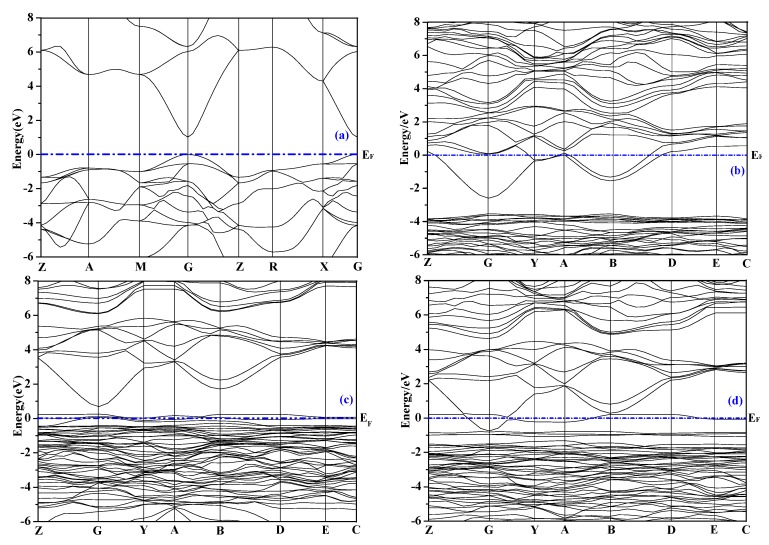
Energy band structure: (**a**) SnO_2_; (**b**) SnO_2_-F; (**c**) SnO_2_-Cu; (**d**) SnO_2_-Cu-F.

The energy band structures of SnO_2_ before and after doping are shown in Figure 2, the 0 eV is selected as the Fermi level (*E*_F_). Considering that the study on the deep level is of little significance, therefore, only the band structure near the Fermi level is given.

Intrinsic SnO_2_ is a wide bandgap semiconductor material with a band gap value of 3.6 eV [9], the band gap value of SnO_2_ calculated in this paper is 1.016 eV, which is smaller than the experimental value because the Generalized Gradient Approximation (GGA) used in this paper underestimates the energy of the excited state electrons in the conduction band, but it does not affect the analysis of the variation trend of the band gap value before and after doping. It can be seen from the figure that the conduction band bottom and the valence band top of each doping system are located at the G point of the Brillouin zone, indicating that the SnO_2_ after doping are still direct bandgap semiconductor materials. Compared with the intrinsic SnO_2_, the energy band curve of the doped SnO_2_ becomes dense and its locality increases. Figure 2b is the energy band structure diagram of the F-doped system, its bandgap value is 0.93 eV. The Fermi level passes through the conduction band, indicating that the F doping is an n-type doping; Compared with the O atom, the F atom only needs to attract one electron to become an F^1-^ion, resulting in an increase in the number of free electrons. Figure 2c is the energy band structure diagram of Cu single-doped system, the Fermi level passes through the top of the valence band, indicating that Cu doping belongs to p-type doping. Compared with the intrinsic SnO_2_, the conduction band of SnO_2_-Cu moves down, and the valence band moves up, and impurity levels (mainly composed of Cu-3d state) are introduced in the forbidden band, so that the carrier concentration is increased, the band gap value is narrowed to 0.45 eV, and the conductivity is enhanced. Figure 2d is the energy band structure diagram of the Cu F co-doped system, the Fermi level passes through the conduction band, indicating that the effect of the donor impurity F atom is stronger than that of the acceptor impurity Cu atom, so that the co-doped system belongs to n-type doping. The complementation of donor impurities and acceptor impurities widens the impurity energy level, increases carrier concentration, narrows the band gap value to 0.06 eV, and reduces the energy required for electrons to transition from valence band to conduction band, therefore, under the joint action of Cu and F atoms, the conductivity of the co-doped system is further enhanced.

#### 3.2.2. Electron Effective Mass and Ionizing Energy of Donor

According to the energy band structure, the electron effective mass at the bottom of the conduction band of SnO_2_-Cu and SnO_2_-Cu-F was calculated by using the electron effective mass formula to further analyze the influence of F atoms on the electrical conductivity [10]. The smaller the electron effective mass, the narrower the band gap, the greater the electron mobility, and the stronger the conductivity.

(3) $$\frac{1}{m_{n}^{*}}=\frac{4\pi^{2}}{h^{2}}{\frac{d^{2}E\left(\kappa \right)}{d\kappa^{2}}}^{}$$

where, $m_{n}^{*}$ is the electron effective mass, *h* is the Planck constant, κ is the wave vector, and E(κ) is the energy of the electron at the corresponding wave vector. It can be seen from the calculation results that the electron effective mass of SnO_2_-Cu-F(0.586m_0_) is less than that of the SnO_2_-Cu(0.740m_0_), indicating that under the synergistic action of Cu and F atoms, the electron effective mass of the co-doped system is reduced, the electron mobility is increased, and the conductivity is enhanced. 

Donor ionization energy refers to the energy required for a valence electron on impurity atoms to be excited into conduction band or valence band to become a free electron, the smaller the value, the easier a valence electron becomes a free electron. The donor ionization energy calculation formula is [11]: $E_{i}=m_{n}^{*}E_{0}/m_{0}\varepsilon_{r}^{2}$, $E_{0}$ is the ionization energy of the ground state electron of hydrogen atom (13.6 eV), *m*_0_ is the mass of the free electron, $\varepsilon_{r}$ is the relative dielectric constant of SnO_2_. According to the formula, the donor ionization energy is proportional to the electron effective mass (other parameters are constant), under the action of F atoms, the donor ionization energy of the co-doped system decreases, and the energy required for valence electrons to become free electrons decreases. Under the same condition, the number of free electrons in the Cu F co-doped system is more than that in the Cu single-doped system, so SnO_2_-Cu-F has stronger conductivity.

#### 3.2.3. Density of States

**Figure 3:**
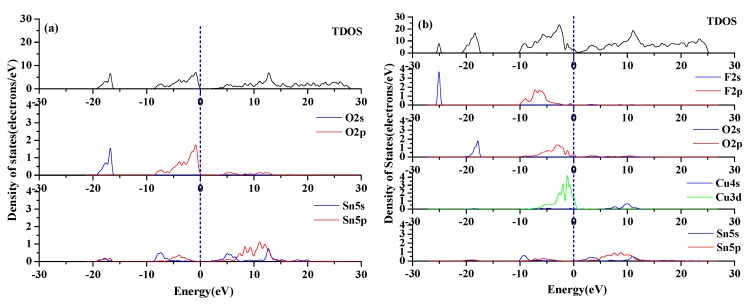
Density of states (**a**) intrinsic SnO_2_; (**b**) SnO_2_-Cu-F.

Density of states can reflect the interaction between doped atoms and other atoms, as well as the formation of chemical bonds in crystals, it is an important parameter for analyzing the electronic structure of materials. Figure 3 shows the density of states of intrinsic SnO_2_ and SnO_2_-Cu-F to analyze the effect of Cu and F co-doping on the electronic structure of SnO_2_, the dotted line represents the Fermi level.

It can be seen from Figure 3 that the total density of states of the co-doped system increases sharply, indicating that the carrier concentration increases after doping. The conduction band of SnO_2_-Cu-F moves to the Fermi level, its locality is enhanced, and the degree of electron sharing is increased. An impurity level peak appears near the top of the valence band (mainly formed by the hybridization of Cu-3d state and O-2p state, which also contains a small amount of F-2p state), these factors together lead to the enhancement of the conductivity of the co-doped system. The pseudogap refers to the distance between the density peaks on both sides of the Fermi level, the longer the distance is, the stronger the covalentity of the chemical bond will be. It can be seen from the Figure 3 that the pseudogap of the SnO_2_-Cu-F is smaller than that of the SnO_2_, which may be the reason why the mechanical properties of SnO_2_-Cu-F are reduced and the toughness is improved. It can be seen from Figure 3b that an impurity peak composed of F-2s state appears in -25ev, which widens the valence band, but is far away from the Fermi level and has little influence on the material properties. Both the Sn-5p orbital and the Cu-3d orbital overlap with the F-2p orbital, indicating that hybridization exists between F atoms, and Cu, Sn atoms, respectively, and Cu-F and Sn-F bonds are formed.

### 3.3. Mechanical Properties

The high hardness and brittleness of SnO_2_ are the main factors affecting the processing properties of AgSnO_2_ contact material. The elastic coefficients of different doping systems are calculated by the same method, and the Young’s modulus, bulk modulus, shear modulus, and Poisson’s ratio are calculated respectively according to the elastic coefficient to analyze their hardness, toughness, and other mechanical properties. The tetragonal phase system has six independent elastic coefficients—C_11_, C_33_, C_44_, C_66_, C_12_, and C_13_—because of the symmetry, the calculation results are shown in Table 2.

The elastic coefficients can be used to judge whether the mechanical structure of the material is stable, and the criterion for determining the mechanical stability of the tetragonal phase crystal system is [12]:

C_11_ > 0, C_33_ > 0, C_44_ > 0, C_66_ > 0, (C_11_ − C_12_) > 0 (C_11_ + C_33_ − 2C_13_) > 0, [2(C_11_ − C_12_) + C_33_ + 4C_13_] > 0 (4)

According to the data in Table 2, the three groups of doping systems all meet the criteria for determining the mechanical stability. The Voigt-Reuss-Hill approximation algorithm [13] was used to calculate the bulk modulus and shear modulus of SnO_2_ under different doping conditions, Hill believed that the results obtained by Voigt and Reuss approximation algorithm were respectively the maximum and minimum values of the elastic modulus of the polycrystal, and the arithmetic average value was closer to the actual elastic modulus of the crystal, so, the calculation formulas of the shear modulus *G* and the bulk modulus *B* are as follows: (the subscripts R and V represent Reuss and Voigt approximation algorithms respectively)

(5) $$G_{H}=\frac{1}{2}(G_{R}+G_{V}),\;B_{H}=\frac{1}{2}(B_{R}+B_{{}_{V}}^{})$$

According to the Voigt approximation algorithm [14], the formulas for shear modulus and bulk modulus are as follows:

(6) $$G_{V}=\frac{1}{15}(2C_{11}+C_{33}-C_{12}-2C_{13})+\frac{1}{5}(2C_{44}+C_{66}),\;\;B_{V}=\frac{1}{9}(2C_{11}+C_{33})+\frac{2}{9}(2C_{13}+C_{12})$$

According to the Reuss approximation algorithm [15], the formulas for shear modulus and bulk modulus are as follows:

(7) $$G_{R}=15{\left[\frac{18B_{V}}{\left(C_{11}+C_{12}\right)C_{33}-2C_{13}^{2}}+\frac{6}{C_{11}-C_{12}}+\frac{6}{C_{44}}+\frac{3}{C_{66}}\right]}^{-1}\;B_{R}=\frac{\left(C_{11}+C_{12}\right)C_{33}-2C_{13}^{2}}{C_{11}+C_{12}+2C_{33}-4C_{13}}$$

The formulas for calculating Young’s modulus (*E*), Poisson’s ratio (*γ*), and hardness (*H*) are:

(8) $$E=\frac{9B_{H}G_{H}}{3B_{H}+G_{H}},\;\gamma =\frac{3B_{H}-2G_{H}}{2(3B_{H}+G_{H})},\;H=\frac{(1-2\gamma )E}{6(1+\gamma )}$$

Table 3 shows the elastic parameters such as bulk modulus (*B*), shear modulus (*G*), Young’s modulus (*E*), Poisson’s ratio *(γ*), and hardness (*H*) of SnO_2_ crystals before and after doping.

The bulk modulus can characterize the strength of the material’s resistance to volume change, and the greater the value, the stronger the resistance to volume change; Shear modulus can measure the strength of the material’s resistance to shear strain; Young’s modulus is a physical quantity that characterizes the tensile or compressive capacity of the material within elastic limits, the larger the value is, the less likely the material is to deform; The hardness can reflect the strength of the wear resistance of the material, and the larger the value, the better the wear resistance of the material and the longer the life. It can be seen from Table 3 that the bulk modulus, shear modulus, Young’s modulus, and hardness of SnO_2_ after doping are reduced compared with intrinsic SnO_2_, indicating that doping impairs the mechanical properties of SnO_2_. Among the three groups of doping systems, SnO_2_-Cu-F has the highest shear modulus, Young’s modulus, and hardness, and its mechanical properties and wear resistance are relatively best, and its service life is also the longest, indicating that the weakening degree of the physical properties of SnO_2_ by Cu F co-doping is less than that by Cu and F single doping.

Pugh believes that the ratio of bulk modulus to shear modulus can be used to determine whether a material is a brittle or ductile material [16], when *B/G* > 1.75, it is considered that the material belongs to ductile material, otherwise, it belongs to brittle material. It can be seen from Table 3 that the B/G values of the three doped systems are all greater than 1.75, in which the B/G value of SnO_2_-Cu-F is the lowest and its toughness is relatively weakest, however, compared with the intrinsic SnO_2_, the toughness of SnO_2_-Cu-F has been greatly improved. Poisson’s ratio can also be used to characterize the brittleness and toughness of materials. If *γ* > 0.26, the material is considered to be a ductile material; the larger the value, the better the ductility of the material [17], it can be seen from the calculation results that the change trend of Poisson’s ratio is consistent with the analysis result of B/G values.

### 3.4. Debye Temperature

Debye temperature is an important parameter to analyze the properties of materials, which relates the mechanical parameters and thermodynamic properties of materials. The higher the debye temperature, the stronger the binding force between atoms, the more stable the crystal structure, the greater the hardness, and the higher the melting point. The calculation formulas of Debye temperature are as follows [18]:

(9) $$\Theta_{D}=\frac{h}{k_{B}}{\left(\frac{3nN_{A}\rho }{4\pi M}\right)}^{1/3}V_{m},\;V_{m}={\left(\frac{2}{3v_{t}^{3}{}_{}}+\frac{1}{3v_{l}^{3}}\right)}^{-1/3}$$

where, *Θ*_D_ is the Debye temperature, *k*_B_, *h*, and *N*_A_ are Boltzmann constant, Planck constant, and Avogadro constant, respectively, *n*, *M*, *ρ* are the number of atoms in the unit cell, the molecular mass, and density of the unit cell, respectively. *V*_m_ is the average wave velocity, *V*_t_ and *V*_l_ are the transverse wave velocity and the longitudinal wave velocity, respectively.

(10) $$V_{t}=\sqrt{G/\rho },\;V_{l}=\sqrt{\left(B+4G/3\right)/\rho }$$

Table 4 shows the calculation results of debye temperature of SnO_2_ under different doping conditions. It can be seen from Table 4 that in the three groups of doping systems, the Debye temperature of SnO_2_-Cu-F is the highest, indicating that the bonding force between atoms is the strongest in the co-doped system, and the structure is the most stable; Compared with the Cu single-doping system and the F single-doped system, the Cu F co-doped system has higher hardness and higher melting point, which is consistent with the previous analysis results.

## 4. Experiment

### 4.1. X-Ray Diffraction Experiment

**Figure 4:**
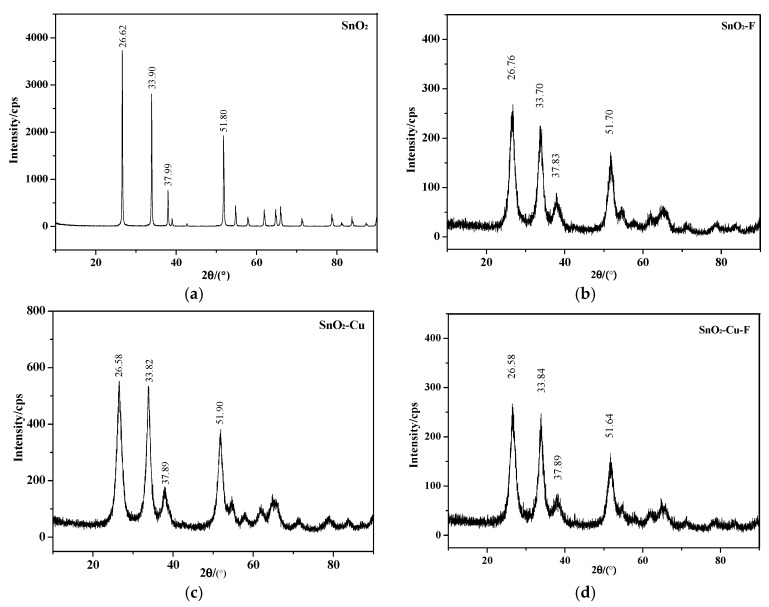
XRD patterns of SnO_2_ powder before and after doping. (**a**) SnO_2_; (**b**) SnO_2_-F; (**c**) SnO_2_-Cu; (**d**) SnO_2_-Cu-F.

Using the Bruker D8 DISCOVER X-ray diffractometer (Bruker Axs, Karlsrube, Germany to analyse the crystal structure of SnO_2_ powder under different doping conditions, the scanning range was 10°–90°, and the scanning speed was 6°/min. Figure 4 shows the results of an X-ray diffraction experiment and gives the angles of four representative diffraction peaks. It can be seen from the figure that the angle positions of the four diffraction peaks of doped SnO_2_ do not change much, but slightly shift to a lower angle compared with the intrinsic SnO_2_. The shift of the XRD peaks is due to the increase of lattice constant caused by the doping atoms. Moreover, there are no diffraction peaks associated with Cu and F atoms, indicating that Cu and F atoms were well doped into the lattice of SnO_2_ during the sol-gel process, and the structure of SnO_2_ is not changed, therefore, the doped SnO_2_ still belongs to tetragonal rutile structure. The intensity of the diffraction peak of SnO_2_ after doping decreases, indicating that doping can reduce the crystallinity of the material, and the reduction of crystallinity will lead to the weakening of hardness and brittleness of the material and the strengthening of toughness.

### 4.2. Wettability Test

**Figure 5:**
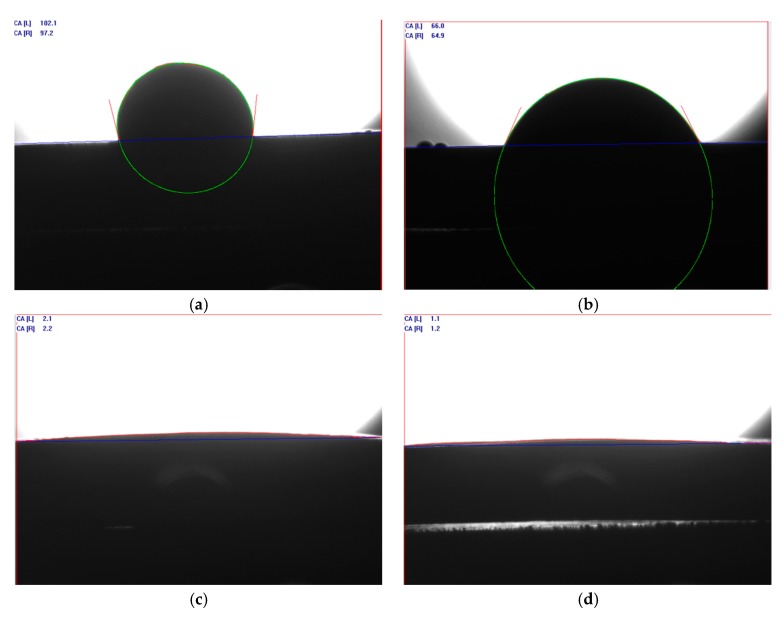
Wetting angle test results: (**a**) SnO_2_; (**b**) SnO_2_-F; (**c**) SnO_2_-Cu; (**d**) SnO_2_-Cu-F.

The wettability between SnO_2_ and Ag liquid is the key factor affecting the contact resistance of AgSnO_2_ contact, the better the wettability, the less likely it is to form SnO_2_ enrichment zone on the contact surface, and the smaller the contact resistance [19]. The wetting angle (θ) can be used to determine whether the wettability is good or bad, and the smaller the wetting angle, the better the wettability between the two phases. Using Sessile Drop Method to measure the wetting angle between different doped SnO_2_ and Ag liquid, during the experiment, the temperature was set to 1050 °C (higher than the melting point of Ag), the temperature was maintained for 0.5 h, and the mass of Ag particles was 0.3 g. The contact angle meter was used to take photos of the cooled samples, and the wetting angles were measured by drawing the contour, baseline, and tangent on the photos. The wetting angle measurement charts are shown in Figure 5, and the wetting angle results are shown in Table 5.

The wetting angle between the intrinsic SnO_2_ and the Ag liquid is 99.65°, the wettability is poor, the wetting angle between the SnO_2_-F sample and Ag liquid is 65.45°, and the wettability is improved. The Figure 5c shows that the Ag particle is spread on the SnO_2_-Cu sample, most of which are melted into the sample, and as the wetting angle is reduced to 2.15°, and the wettability is greatly improved which is consistent with the study of the literature [3]. According to Figure 5d, Ag particle is further spread on the SnO_2_-Cu-F sample, and the wetting angle is 1.15°, indicating that Cu F co-doping can further improve the wettability between SnO_2_ and Ag liquid, so that it is difficult for SnO_2_ to precipitate from the Ag liquid to form the SnO_2_ enrichment zone. And the contact resistance will be reduced, which is consistent with the contact resistance test results.

### 4.3. Hardness, Conductivity, and Electrical Contact Simulation Experiment

The hardness, conductivity, and electrical contact simulation experiments were carried out on the prepared AgSnO_2_ contact, AgSnO_2_-Cu, contact and AgSnO_2_-Cu-F contact. The hardness, conductivity, contact resistance, arc energy, and arc duration were measured to verify the theoretical analysis. The hardness of the samples were measured by using a HXD-1000TM digital microhardness tester (Shanghai Optical Instrument Factory, Shanghai, China). The Sigmas cope SMP10 metal conductivity tester (Fischer, Bad Salzuflen, Germany) was used to measure the conductivity of the samples, and each sample was measured 5 times, the average value was taken as the final result. The prepared contact samples were cut into contact points with a diameter of 4.5 mm and a thickness of 3.5 mm, JF04C electrical contact material test system was used for electrical contact simulation experiment. The experimental parameters: current was DC13A, voltage was DC24V, contact pressure was 86 cN. We prepared three samples for different doping types to be tested. Each sample was subjected to 25,000 electrical contact simulation experiments the contact resistance, arc energy, and arc duration were measured every 100 times, and the average value was taken as the final result. The experimental results are shown in Table 6.

It can be seen from Table 6 that the hardness and contact resistance of the contact after doping are reduced, and the electrical conductivity is increased, indicating that doping can improve the toughness and electrical conductivity of the contact. The hardness of the AgSnO_2_-Cu-F contact (82.03 HV) is greater than the hardness of the AgSnO_2_-Cu contact (76.12 HV), indicating that the mechanical properties and wear resistance of AgSnO_2_-Cu-F contact are relatively better, and its service life is longer, which is consistent with the theoretical analysis of mechanical properties. The conductivity of the AgSnO_2_-Cu-F contact is 31.20 mS⋅m^−1^, which is greater than the conductivity of the AgSnO_2_-Cu contact (28.87 mS⋅m^−1^), indicating that the conductivity of the contact is further enhanced after the introduction of the F atoms, which is consistent with the analysis results of the electronic structure. The contact resistance of the AgSnO_2_-Cu-F contact (1.048 mΩ) is smaller than the contact resistance of the AgSnO_2_-Cu contact (1.502 mΩ), which is consistent with the wettability test results. Arc duration and arc energy can be used to measure the arc corrosion resistance of the contact material. The smaller the values are, the stronger the arc erosion resistance is. It can be seen from Table 6 that the arc duration (2.76 ms) and arc energy (190.6 mJ) of AgSnO_2_-Cu-F contact are both smaller than those of AgSnO_2_ contact, which indicates that Cu F co-doping can enhance the arc corrosion resistance of the contact.

**Figure 6:**
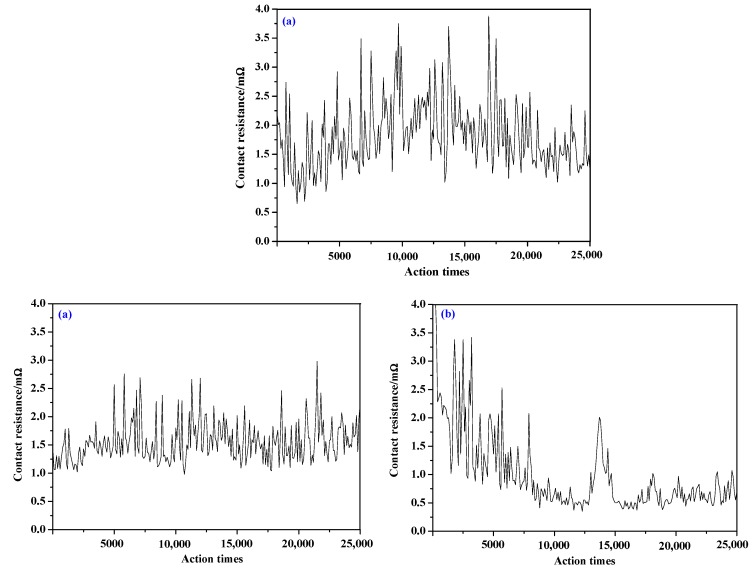
Contact resistance: (**a**) AgSnO_2_; (**b**) AgSnO_2_-Cu; (**c**) AgSnO_2_-Cu-F.

Figure 6 shows the test results of contact resistance of AgSnO_2_ contact, AgSnO_2_-Cu contact, and AgSnO_2_-Cu-F contact. It can be seen from the figure that the change trend of the contact resistance of the AgSnO_2_ contact is first increased and then decreased, and its range of variation is the largest and the stability is poor. The contact resistance of AgSnO_2_-Cu contact fluctuates around the average value, and the stability is relatively better. The contact resistance of AgSnO_2_-Cu-F contact decreases gradually with the increase of action times, and finally remains relatively stable. The initial contact resistance values of AgSnO2-Cu-F are larger than those of AgSnO_2_ contact and AgSnO_2_-Cu contact, which may has some influences on the initial contact of switching devices. However, after 7000 electric contact simulation experiments, the contact resistance of AgSnO_2_-Cu-F becomes low and stable, which meets the requirements of ideal contact material. And the fluctuation around 14,000 times may be caused by the sudden change of contact pressure, and this problem can be solved by adjusting the parameter of contact pressure.

## 5. Conclusions

The first-principles method based on density functional theory was used to analyse the crystal structure, electronic structure, mechanical properties, and debye temperature of pure SnO_2_, F doped SnO_2_, Cu doped SnO_2_, and Cu F co-doped SnO_2_. The SnO_2_ powders with different additives were prepared by using the sol-gel method. AgSnO_2_ contacts with different additives were prepared by using the powder metallurgy method, and a series of experiments were carried out. The results show that although the co-doping of Cu and F increases the lattice constant and volume of SnO_2_, it does not change its structure, so the doped SnO_2_ still belongs to the tetragonal phase rutile structure. And the doping formation energy of SnO_2_-Cu-F is the smallest, and the co-doping is easier to realize and the structure is more stable than SnO_2_-Cu and SnO_2_-F. Cu F co-doping can further narrow the band gap, reduce the electron effective mass and donor ionization energy, increase the electron mobility, and further enhance the conductivity of SnO_2_. The wetting angle between the SnO_2_-Cu-F and the Ag liquid is the smallest, indicating that Cu F co-doping can further improve the wettability between SnO_2_ and Ag liquid, and reduce the contact resistance of the contact. Experiments show that the AgSnO_2_-Cu-F contact has the best electrical properties, its conductivity is 31.20 mS⋅m^−1^, and the contact resistance is 1.048 mΩ. Among the three groups of doping systems, the SnO_2_-Cu-F has the highest shear modulus, Young’s modulus, hardness, and Debye temperature; its mechanical properties and wear resistance are relatively best, and the melting point is also the highest. The hardness of the AgSnO_2_-Cu-F is 82.03 HV, which is smaller than that of the AgSnO_2_ (117.1 HV), indicating that the co-doping can improve the processing performance of the contact.

## Figures and Tables

**Table 1:** Lattice constant, volume, and doping formation energy of SnO_2_ under different doping conditions.

Parameter	SnO_2_	SnO_2_-F	SnO_2_-Cu	SnO_2_-Cu-F
a/Å	4.737	4.963	4.861	4.892
b/Å	4.737	4.970	4.845	4.909
c/Å	3.186	3.354	3.250	3.266
V/Å^3^	286.0	330.8	306.2	313.6
E_f_/eV	-	−4.39	−1.97	−8.89

**Table 2:** The elastic coefficients of intrinsic SnO_2_ and different doping systems.

Doping Model	C_11_/GPa	C_12_/GPa	C_13_/GPa	C_33_/GPa	C_44_/GPa	C_66_/GPa
SnO_2_	204.4	131.2	114.3	357.0	86.90	177.6
SnO_2_-F	175.8	110.3	118.3	169.8	66.26	65.62
SnO_2_-Cu	169.1	126.5	116.4	175.1	50.25	49.75
SnO_2_-Cu-F	182.5	108.9	119.7	177.2	66.10	69.98

**Table 3:** Bulk modulus (GPa), shear modulus (GPa), Young’s modulus (GPa), poisson’s ratio *γ*, bulk modulus to shear modulus ratio (*B/G*), as well as Hardness (HV), of SnO_2_ before and after doping.

Doping Model	*B*	*G*	*E*	*γ*	*B/G*	*H*
SnO_2_	161.5	96.07	219.3	0.2517	1.681	14.50
SnO_2_-F	143.3	65.76	171.1	0.3010	2.179	8.723
SnO_2_-Cu	149.2	53.89	144.3	0.3388	2.768	5.792
SnO_2_-Cu-F	146.9	70.45	182.2	0.2933	2.085	9.706

**Table 4:** Density *ρ* (g/cm^3^), transverse wave velocity *V*_t_, longitudinal wave velocity *V*_l_, average wave velocity *V*_m_ (m/s), and debye temperature *Θ*_D_ (k) of SnO_2_ under different doping conditions.

Doping Model	*ρ*	*V*_t_	*V*_l_	*V*_m_	*Θ*_D_
SnO_2_-F	4.24	3661	7381	4399	484.5
SnO_2_-Cu	4.02	3938	7415	4110	452.4
SnO_2_-Cu-F	4.04	4176	7721	4660	513.2

**Table 5:** The wetting angles between different doping systems and Ag liquid.

Doping Model	SnO_2_	SnO_2_-F	SnO_2_-Cu	SnO_2_-Cu-F
Wetting angle (θ/°)	99.65	65.45	2.15	1.15

**Table 6:** Experimental results.

Contact	Hardness (HV)	Conductivity (mS⋅m^−1^)	Contact Resistance (mΩ)	Average Arc Energy (mJ)	Average Arc Duration (ms)
AgSnO_2_	117.1	26.44	1.814	215.3	3.34
AgSnO_2_-Cu	76.12	28.87	1.502	184.2	2.51
AgSnO_2_-Cu-F	82.03	31.20	1.048	190.6	2.76
